# Domain Adversarial Convolutional Neural Network Improves the Accuracy and Generalizability of Wearable Sleep Assessment Technology

**DOI:** 10.3390/s24247982

**Published:** 2024-12-14

**Authors:** Adonay S. Nunes, Matthew R. Patterson, Dawid Gerstel, Sheraz Khan, Christine C. Guo, Ali Neishabouri

**Affiliations:** 1 ActiGraph LLC, Pensacola, FL 32502, USA; adonay.s.nunes@gmail.com (A.S.N.); matthew.patterson@theactigraph.com (M.R.P.); dawid.gerstel@theactigraph.com (D.G.); christine.guo@theactigraph.com (C.C.G.); 2 McGovern Institute for Brain Research, Massachusetts Institute of Technology, Cambridge, MA 02139, USA; skhan7@mgh.harvard.edu; 3 Department of Radiology, Massachusetts General Hospital, Harvard Medical School, Boston, MA 02115, USA; 4 Athinoula A. Martinos Center for Biomedical Imaging, Massachusetts General Hospital, Harvard Medical School, Massachusetts Institute of Technology, Boston, MA 02129, USA

**Keywords:** sleep, accelerometer, wearable, deep learning

## Abstract

Wearable accelerometers are widely used as an ecologically valid and scalable solution for long-term at-home sleep monitoring in both clinical research and care. In this study, we applied a deep learning domain adversarial convolutional neural network (DACNN) model to this task and demonstrated that this new model outperformed existing sleep algorithms in classifying sleep–wake and estimating sleep outcomes based on wrist-worn accelerometry. This model generalized well to another dataset based on different wearable devices and activity counts, achieving an accuracy of 80.1% (sensitivity 84% and specificity 58%). Compared to commonly used sleep algorithms, this model resulted in the smallest error in wake after sleep onset (MAE of 48.7, Cole–Kripke of 86.2, Sadeh of 108.2, z-angle of 57.5) and sleep efficiency (MAE of 11.8, Cole–Kripke of 18.4, Sadeh of 23.3, z-angle of 9.3) outcomes. Despite being around for many years, accelerometer-alone devices continue to be useful due to their low cost, long battery life, and ease of use. Improving the accuracy and generalizability of sleep algorithms for accelerometer wrist devices is of utmost importance. We here demonstrated that domain adversarial convolutional neural networks can improve the overall accuracy, especially the specificity, of sleep–wake classification using wrist-worn accelerometer data, substantiating its use as a scalable and valid approach for sleep outcome assessment in real life.

## 1. Introduction

Sleep is a fundamental biological process essential for physical and mental health. Insufficient or poor-quality sleep has been linked to a wide range of adverse health outcomes, including cardiovascular disease, metabolic disorders, and mental health conditions [1,2,3]. Despite decades of research, many aspects of sleep physiology, sleep disorders, and their relationships with chronic conditions remain poorly understood. The ability to monitor sleep accurately and cost-effectively across large populations is critical for advancing research, diagnosis, and treatment of sleep-related disorders.

Sleep monitoring refers to the process of measuring and analyzing sleep patterns, including the duration, quality, and timing of sleep, as well as periods of wakefulness. Polysomnography (PSG), the clinical gold standard for sleep monitoring, provides detailed insights into physiological states using sensors to record brain activity, muscle movements, and other signals. However, its resource-intensive nature and reliance on controlled environments make PSG unsuitable for large-scale or long-term monitoring. To address these limitations, wearable devices such as wrist-worn accelerometers have gained popularity for tracking sleep in real-world conditions. These devices offer a practical and non-intrusive solution, enabling continuous data collection in free-living environments [4].

Since the introduction of wrist-worn actigraphy in the early 1980s [5], several generations of automated scoring algorithms have been developed [6]. Earlier wrist-worn accelerometers only reported data in the form of *counts* [7], which are a way to quantify movement over a given period. Early sleep scoring algorithms were thus developed to use these counts as their input. These algorithms range from simple logistic regression models, to more sophisticated machine learning and deep learning techniques [5,8,9,10,11]. Later, wearable devices capable of recording raw accelerometer data enabled the use of heuristic and machine learning approaches for analyzing raw acceleration, enhancing the development of sleep scoring algorithms in the 2010s [12,13]. Despite these advancements, one persistent challenge in sleep scoring via wrist-worn accelerometry is the balance between specificity and sensitivity. High specificity in detecting wake periods often comes at the expense of sensitivity, leading to compromised overall accuracy in sleep–wake classification.

The primary objective of this study is to develop a model for detecting sleep and wake states from activity counts that is both accurate and generalizable. Sleep–wake classification models often face a trade-off between specificity (accuracy in detecting sleep) and sensitivity (accuracy in detecting wake). This trade-off is particularly problematic for sleep-disordered patients, who experience more frequent wake episodes compared to healthy individuals. While high specificity is relatively easy to achieve due to the predominance of sleep epochs, achieving high sensitivity without compromising overall accuracy remains a significant challenge [14].

Existing machine learning and deep learning models for sleep classification are prone to overfitting to the specific characteristics of their training datasets. This issue is exacerbated by the use of activity counts, which vary depending on the computation method. Although efforts have been made to standardize these metrics [7], current models often fail to generalize to new datasets with different activity count methods [15]. This lack of generalizability limits the practical application of these models in large-scale population studies and across diverse user groups.

Given the rise in demand for sleep monitoring with wearables, it is important to refine and develop models to obtain better sleep–wake detection accuracy and improve the estimation of outcome measures such as sleep efficiency (SE, sleep time divided by time in bed) and wake-after-sleep-onset (WASO; total minutes of wake during the in-bed sleep interval after sleep onset). Although many datasets of PSG-validated sleep data have been described [6], few are publicly accessible. Currently, the Multi-Ethnic Study of Atherosclerosis (MESA) dataset [16,17] is the largest dataset with concurrent PSG and actigraphy. In the MESA dataset, raw acceleration is not available, activity counts are the only available data from the wrist-worn accelerometers. In this study, we first trained a convolutional neural network (CNN) model with the MESA dataset and achieved good performance. Then, we improved this model using a domain adversarial convolutional neural network (DACNN) to allow it to generalize to a validation dataset based on a different type of activity count computed from an open-source package [7].

Machine learning or deep learning approaches are powerful data science techniques and have substantially improved the accuracy of feature detection in many disciplines, such as imaging and voice recognition. These approaches, however, are susceptible to the risk of overfitting. The models may latch on to particularities in the training dataset that the end-user would deem unimportant, and their performance would suffer on unseen datasets. Model generalization is even more challenging in using accelerometry data for sleep–wake classification. Many big sleep datasets based on accelerometry only retained the activity “counts” as the source data. Activity “counts” are derived from raw acceleration data using non-standard algorithms, many of which are black-box approaches proprietary to the manufacturers. While efforts have been made to document “counts” and make them open-source [7], differences in how activity counts are computed pose another challenge for models trained using one activity count method to generalize to another dataset using another activity count method. This challenge has been observed in previous work applying deep learning sleep algorithms to unseen datasets using different counts [15].

Improving model generalizability is crucial to the application of machine learning and deep learning models to real-world problems, especially in medicine. The machine learning community has developed many techniques for overcoming these issues such as using dropout [18], max pooling [19], and early stopping [20]. One approach is to use a domain adversarial neural network [21,22] to train a model with one dataset but enforcing the features to be indistinguishable from a domain dataset. A domain adversarial approach is particularly effective in improving generalizability to a target dataset when the datasets contain non-linear differences that cannot be corrected by unit-scaling the observations. This would be the case for datasets where different activity counts are used which have different sensitivities in detecting activity counts [23].

To address these challenges, this study builds on the existing body of work in sleep monitoring by making the following contributions: We develop a convolutional neural network (CNN) model specifically optimized for detecting wake states with high sensitivity, which is critical for studying sleep-disordered populations. A domain adversarial convolutional neural network (DACNN) approach is employed to improve the model’s ability to generalize across datasets with different activity count computation methods. We evaluate the model’s performance in estimating key sleep metrics. By focusing exclusively on activity counts, we prioritize scalability and applicability in large-scale, free-living studies.

This paper is structured as follows: Section 2 details the datasets and modeling approach; Section 3 presents the performance evaluation; and Section 4 discusses the implications, limitations, and future directions of this work.

## 2. Materials and Methods

### 2.1. Datasets

The MESA sleep studies [16,17] were conducted on 2237 participants using an Actiwatch Spectrum device (Philips Respironics) which recorded activity counts at 30-s epochs. Overall, 15.0% of individuals had severe sleep-disordered breathing (SDB) (apnea–hypopnea index [AHI] ≥ 30); 30.9% short sleep duration (<6 h); 6.5% poor sleep quality (sleep efficiency < 85%); and 13.9% had daytime sleepiness [16]. Polysomnography (PSG) was recorded simultaneously using a Compumedics Somté System (Compumedics Ltd., Abbotsville, Australia). Institutional Review Board approval was obtained at each study site and written informed consent was obtained from all participants. Sleep stages and EEG (cortical) arousals were scored according to published guidelines [24].

The Newcastle (NC) dataset [13,25] is composed of 28 participants that participated in a sleep study with concurrent PSG and actigraphy. Of these, 27 of the participants wore two accelerometers, one on each wrist, except while 1 participant only wore an accelerometer on one wrist. There are 55 records in total that contain sleep stage information as well as three channels of raw acceleration. The acceleration data were recorded using a triaxial accelerometer (GENEActiv, Activinsights Ltd, Kimbolton, UK). The accelerometers collected data at a sampling rate of 85.7 Hz with a range of ±8g. PSG was recorded using an Embletta® (Denver) system. All respiratory events and sleep stages were scored according to standard criteria [26]. A total of 20 of the participants had at least one sleep disorder. The sleep disorders included idiopathic hypersomnia, restless leg syndrome, sleep apnea, narcolepsy, sleep paralysis, nocturia, obstructive sleep apnea, RBD, parasomnia and insomnia. The methods were performed in accordance with relevant guidelines and regulations and approved by the NRES Committee North-East Sunderland ethics committee (12/NE/0406).

### 2.2. Data Preprocessing

The MESA dataset contains activity counts aggregated over 30 s windows as well as the PSG labels over 30 s periods. We used these data directly in this study by aligning count data with PSG labels based on timestamps. Participants without a full night of concurrent PSG and actigraphy, with less than 3 h of data, or with more than 16 h of data, were discarded, as in [9]. The resultant number of recordings used in the MESA was 1817 participants.

The NC dataset is composed of raw acceleration data and 30 s window PSG labels. The acceleration data in the NC dataset were preprocessed using the *GGIR* package [27] and raw triaxial accelerometer data were calibrated relative to the gravitational acceleration and temperature [28]. All subjects in the NC dataset were kept.

Since raw acceleration data were not available for the MESA dataset, the model development only used activity counts as input. In the case of the MESA dataset, activity counts are provided in the dataset, although the algorithm used to derive them from the raw accelerometer data is not publicly available. In the case of the NC dataset, raw accelerometer data were down-sampled to 40 Hz (taking the mean in 25 ms windows), and then activity counts were obtained using the open-source package *agcounts* [7]. The PSG data collected in the study was only used as the ground truth and was not given as an input to the model.

Sleep onset latency was not included as a sleep endpoint in this work because sleep diary information was not collected with the NC dataset and sleep onset latency based on acceleration alone has been shown to be unreliable [29].

### 2.3. Model Architecture

**Figure 1:**
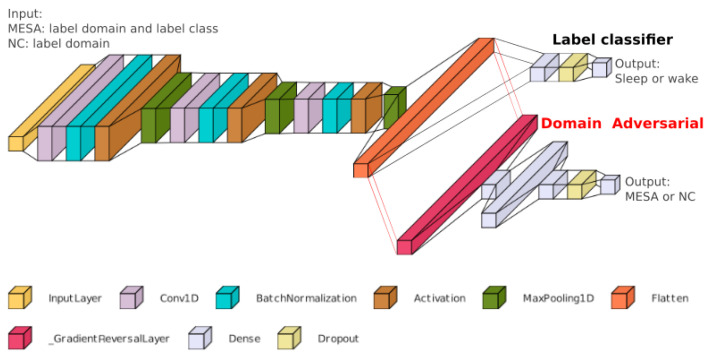
Model architecture. The DAsleepCNN model is composed of modules of a convolutional layer, batch normalization, and max pooling; after three modules the output is flattened and sent to a label classifier where it predicts sleep–wake labels for the MESA dataset, and to a domain adversarial classifier that classifies the dataset domain of the input. For MESA samples, the input labels are the dataset label and sleep–wake label, for NC samples only the dataset label is provided. For inference, the domain adversarial component is not used, only the label classifier.

The input to our models is a time series of accelerometer counts. We first trained a model with three 1D convolutional layers, each followed by batch normalization, activation, and max pooling (Figure 1). Batch normalization was used to regularize the weights of the kernels, speed up convergence, and normalize the features from the convolutions to make them more robust to changes in magnitude. The max-pooling layers reduce the number of parameters needed to be trained by half, but at the same time expand the receptive field by two and posterior layers extract features that expand further in the time dimension.

The model performed well on the MESA dataset on which it was trained but showed limited accuracy when dealing with data from the NC dataset. Although the MESA dataset is much larger than the NC dataset, differences were observed in the mean values of activity counts during sleep and wake between the two datasets. This warranted using a domain adversarial network to force the model to derive features from the MESA dataset that are not distinguishable from the NC dataset, but without providing any labels from the NC dataset to the model during training. Figure 1 shows the model architecture, where after the third max-pooling layer the network is split in two heads: a label classifier and a domain classifier. The noDACNN network only includes the label classifier head, whereas the DACNN network includes the domain adversarial head. The classifier predictor is composed of a fully connected layer, a dropout, and an output layer. The domain predictor has a gradient reversal operator, a fully connected layer, and an output layer. The labels for the classifier predictor are MESA sleep–wake, but not NC labels. For the domain predictor, the labels are MESA/NC, thus this predictor tries to identify the provenance of the sample. When performing backpropagation, the loss from the domain predictor is inverted by the gradient reversal operator and penalizes the network if the prediction is correct; thus, the model is incentivized to extract features that are the least distinguishable between dataset domains.

We explored two input lengths. First, an input length of 25 min prior to the current epoch, the current epoch, and 25 min after the current epoch was used. Previous studies demonstrated that this is an ideal length for the models to have adequate temporal context [9,11]. The second input was the past 25 min, the current epoch, and only 1 min of future activity. The rationale for one minute was to provide enough feature context to assess if the current activity over the 30 s epoch was transient or stable over two consecutive time points. Given that each epoch is 30 s, the input length in the first case was 101 samples and the second was 53 samples. In the MESA dataset, a split of 65-20-15% was used for training, validation, and testing. The NC dataset was only used for validating the results.

### 2.4. Model Performance

The model performance was assessed by measuring the accuracy in predicting the correct sleep–wake labels. The confusion matrix and AUC results are reported for the best-performing models for the MESA and NC datasets. Given that the NC dataset was not used for training the models, we report further metrics averaged across subjects and compare them with other algorithms. The algorithms compared are z-angle [13], Cole [8], and Sadeh [10].

The z-angle relies on findings periods of constant wrist angle extracted from raw accelerometer data to detect sleep. Cole is based on the absence of movement, as measured by activity counts. Sadeh relies on 4 features extracted from counts that are then combined using linear discriminant analysis.

Except for the z-angle, all of the algorithms were rescored [5]. Rescoring is a technique developed to enhance specificity in sleep–wake detections by count-based algorithms [5,30]. There are five rescoring rules, which are after at least four epochs scored as wake, the next sleep epoch is relabeled as wake; after at least ten epochs scored as wake, the next three sleep epochs are relabeled as wake; after at least fifteen epochs scored as wake, the next four sleep epochs are relabeled as wake; six epochs or less scored as sleep surrounded by at least ten epochs, before or after, scored as wake are rescored as wake; and then an epoch or less scored as sleep surrounded by at least 20 epochs, before or after, scored as wake are rescored as wake. The metrics used were sensitivity (the percentage of correct sleep), specificity (the percentage of correct wake), precision (the percentage of detected sleep that was correct), and F1 (the harmonic mean of sensitivity and precision). In addition, we computed two sleep endpoints: WASO and SE. The root mean squared error (RMSE) was used to measure the algorithm error in estimating the sleep endpoints.

## 3. Results

In total, four models were trained with an input of the past 25 and future 25 min (25 + 25) or past 25 and future 1 min (25 + 1), and with a domain adversarial (DACNN) network or without it (noDACNN) (see Table 1).

**Figure 2:**
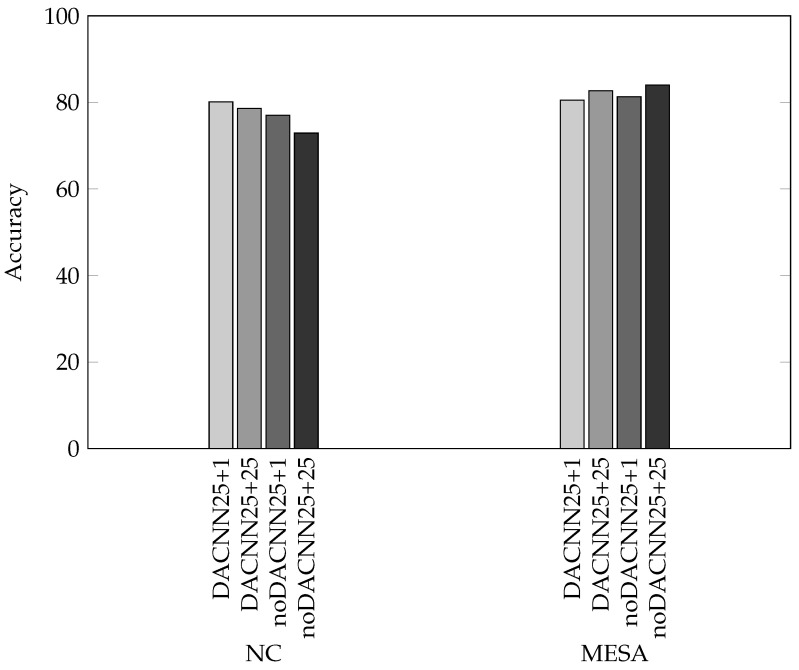
Model accuracy. The accuracies of the four models presented are plotted for the NC and MESA datasets. For MESA, the highest accuracy was achieved by noDACNN25+25. However, this model had a marked drop in performance when applied on NC, showing poor generalizability. DACNN25+1, on the other hand, had a high accuracy on NC, which crucially was on par with its performance on MESA.

As expected, the domain adversarial CNNs performed better than noDACNN for the NC dataset, as the DA penalized convolutional features that are distinguishable between the datasets; thus, the model generalizes better to the target dataset. In the MESA dataset, the noDACNN models performed better, this is also expected, as the features extracted fit the data better without having to be generalized to another dataset. Interestingly, on the NC dataset, the shorter input length of past 25 + future 1 min performed better, with an accuracy of 80.1; and on the MESA dataset, the longer input length of past 25 + future 25 min performed better, with an accuracy of 84.3%. Figure 2 shows the accuracies for different models and datasets.

**Figure 3:**
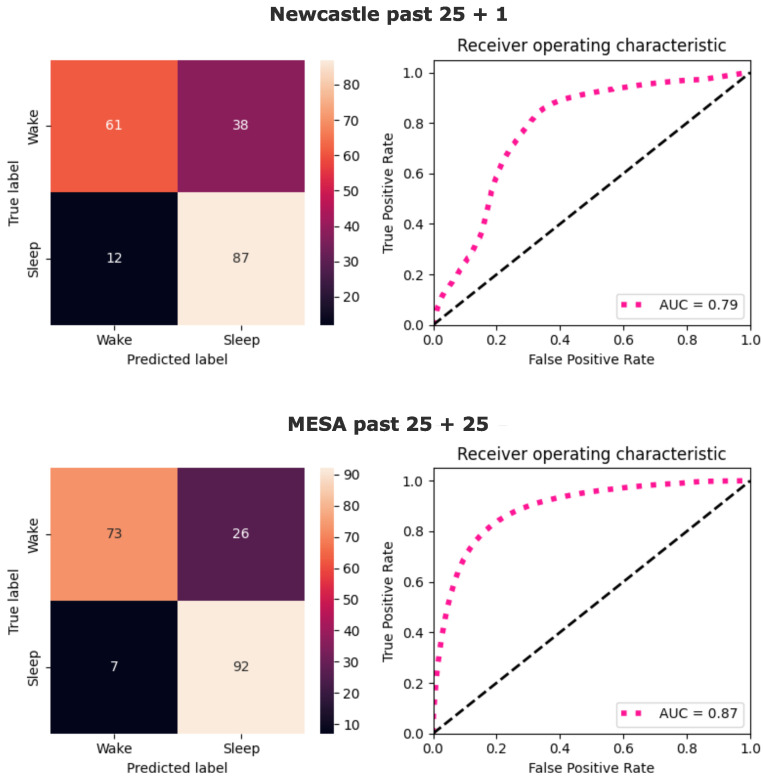
Confusion matrices and ROC-AUC. On top, the confusion matrix and ROC-AUC are plotted for the NC dataset using the best-performing model with input past 25 + 1. On the bottom, the same plots are shown for the MESA dataset with the model input of the past 25 + 25.

The confusion matrices and the receiver operating characteristic (ROC) for the best models for both datasets are shown in Figure 3. The sensitivity in detecting sleep was 87% and 92%, and specificity was 61% and 73%, for the NC and MESA datasets, respectively. On the NC dataset, the AUC reached 0.79, whereas on the MESA it was 0.87. The ROC-AUC curve on the NC dataset was skewed toward a higher false positive rate at the low true positive rate quadrant, as compared to the MESA ROC-AUC curve (Figure 3). The false positive rate indicates the probability of a negative sample (wake) being classified as positive (sleep). This indicates that some samples have a high probability of belonging to a class, but their class prediction is not correct.

**Figure 4:**
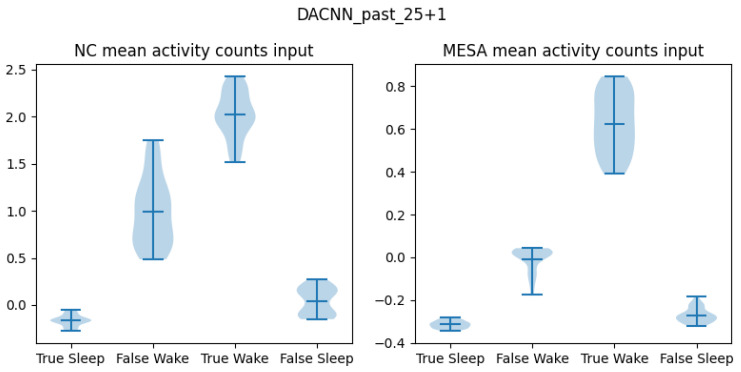
Average input values for correct and incorrect predictions in the datasets. The violin plots show the mean and 25th and 75th interquartile ranges for true and false predictions for the best-performing models. The left represents predictions from the NC dataset and the right from the MESA dataset.

Given the ROC-AUC results, we further examined the average values of inputs that were classified correctly and incorrectly (Figure 4). For both datasets, the mean activity counts of true sleep predictions are lower than the mean of true wake predictions. This is expected as the amount of movement is typically minimal during true sleep periods, while it is likely higher during true wake periods. In the MESA dataset, the activity range of the false wake predictions are close to the range of true sleep, and the mean is below zero. Interestingly, in the NC dataset, the mean activity counts of false wake predictions are above zero and the 75th percentile overlaps with the 25th percentile of true wake. This indicates that on average, sleep periods classified as wake have higher activity than sleep periods classified correctly; similarly, false sleep predictions have lower activity count than wake periods correctly classified.

Since the NC dataset was not used for training the model, it remains a validation dataset that can be used to compare the performance across sleep algorithms (Table 2). The best-performing model, DACNN25+1, outperforms the other deep learning models across almost all metrics in the NC dataset. Only noDACNN25+1 provides higher sensitivity, but at the expense of much lower specificity and overall accuracy than the best model. DACNN25+1 also performs much better than traditional algorithms. Again, due to the trade-off between sensitivity and specificity, while some algorithms, e.g., Sadeh rescored, showed a high specificity of 75.5% but a sensitivity of 61.9%, the best DACNN model demonstrated a balance between sensitivity and specificity, achieving the highest overall accuracy and F1. Importantly, DACNN25+1 also provides the best estimation of sleep outcome measures, with the lowest mean error and root mean squared error as compared to all other models and algorithms.

## 4. Discussion

In this study, we implemented convolutional neural networks to detect sleep–wake from wrist accelerometer data. We used two datasets: one with activity counts already provided by the manufacturer and another where activity counts were computed from the raw acceleration data using an open-source package. Given the difference between the datasets, particularly in the derivation of activity counts, we used a domain adversarial CNN to extract features from the large dataset that were generalizable to the smaller dataset. Two input lengths were explored to give the model a temporal context from which to estimate the class probability of samples. Using the NC dataset as the validation dataset, the top-performing model was the domain adversarial CNN with the past 25 min of activity counts and 1 min of future activity counts. For the MESA, the original large dataset used for training the model, the CNN without the domain adversarial component and with the past and future 25 min of activity counts performed the best. The performance of these two models was superior to previous sleep–wake classification algorithms using the NC dataset as a validation dataset.

While raw accelerometer data can potentially contain more information useful for discriminating wake from sleep, activity count algorithms play a crucial role in sleep research, which is likely to continue to be the case [8,10]. In addition, raw acceleration data are not provided by some wearable manufacturers (Philips) and are not available in many open-source datasets (MESA, STAGES). It is important to recognize that activity counts are not created equal; even derived from the same raw accelerometer data, activity counts might differ due to preprocessing steps such as filter parameters or the precision of the quantization process for estimating the count. These differences can affect the generalizability of algorithms trained across datasets using different activity count methods. It is apparent that the ActiGraph activity counts in the NC dataset have on average much higher values than the Philips activity counts in the MESA datasets (Figure 4). Previous research has shown a non-linear relationship between Philips and ActiGraph activity counts, with the latter being greater when physical activity is higher than 20.

Deep learning models showed different performance with the MESA and NC datasets. Although the CNN model with the past and future 25 min achieved an accuracy of 84.2% and outperformed previous studies that used the same input length, 8, 10, the models did not generalize well to NC data based on different activity counts. The differences in mean activity counts between datasets warranted the use of a domain adversarial neural network, as previous studies have demonstrated them to be useful in extracting features that are generalizable to a validation dataset [31,32,33,34]. When using a domain adversarial network, the model not only performed much better in terms of classification accuracy but also better in terms of sleep outcome estimation. While classification accuracy is important, sleep outcomes are the specific metrics used to assess sleep in clinical trials and care. As shown in Table 1, the DACNN with the input of past 25 + 1 min had the best performance in terms of accuracy, F1, WASO error, and SE error. The results suggest that one minute of future activity counts provides enough context for the model to decide if the current activity is stable or transient. Overall, this model outperforms all the previous algorithms used on this validation dataset, as reviewed by Patterson et al. [15].

There are limitations that do not seem feasible to overcome. As shown in Figure 4, there are situations where participants are awake but there is no movement, and in other instances there is movement but the participant is asleep. This clearly drives the predictability in the NC dataset lower, potentially because half of the participants have a sleep disorder. In the MESA dataset, given the large sample size, these situations may be averaged out. Nonetheless, the performance of models did not increase beyond the reported accuracy of 84.3% in the MESA dataset, suggesting that there is a limit to how well sleep–wake can be predicted from accelerometer-only data. Another limitation is that only two sets of combinations of input lengths were tested; future research should further examine the contributions of different input lengths to model performance.

Recent technological development to incorporate physiological sensing capabilities into wearables holds much potential to increase the accuracy of sleep-stage detection from accelerometer-only devices [14]. Current photoplethysmography (PPG)-based devices must be charged every few days at best, whereas accelerometer-only devices can go for a month or longer on one charge. This is a critical usability aspect for certain patients, for whom charging can be difficult to remember or perform.

Despite intrinsic challenges in sleep–wake classification compared to PSG, a wearable device with an accelerometer allows continuous monitoring of sleep without the need for a complex setup or having to be moved to a sleep lab. The models we present prove that good levels of accuracy can be achieved, and it can reliably estimate sleep endpoints with a margin of error of a few minutes or less than 9%, depending on the unit of the measure.

## Figures and Tables

**Table 1:** Four models trained.

Model	Past 25 min + Future 25 min	Past 25 min + Future 1 min
Without DA	noDACNN25+25	noDACNN25+1
With DA	DACNN25+25	DACNN25+1

**Table 2:** Performance metrics for developed models and prior algorithms. acc: accuracy; sens: sensitivity; spec: specificity; prec: precision; RMSE: root mean squared error; MAE: mean absolute error; CI: confidence interval.

Model	acc	sens	spec	prec	F1	WASO	RMSE	MAE	CI Width	SE	RMSE	MAE	CI Width
${\mathrm{DACNN}}_{25+1}$	80.1	83.9	57.6	81	81.7	159.3	80.9	48.7	309.1	71.8	11.8	8.0	45.7
${\mathrm{DACNN}}_{25+25}$	78.6	81.1	55.7	79.9	79.5	165.2	83.2	54.8	323.8	70.6	12.5	9.1	49.2
${\mathrm{noDACNN}}_{25+1}$	77	84.9	48.5	77.2	80.1	137.1	102.8	76.5	353.4	75.6	17.7	13.5	57.1
${\mathrm{noDACNN}}_{25+25}$	72.9	68.9	68.3	81.3	73.2	231	94.1	57.2	331.5	59	13.3	9.1	46.1
Previous Algorithms													
Z-angle	76.2	83.6	47.5	76.2	79.1	140.8	91.1	57.5	325.3	75	13.0	9.3	46.2
Cole rescored	71.9	66.1	73.6	82.6	72.2	247.5	105.8	86.2	322.5	56.3	18.4	15.4	51.7
Sadeh rescored	69.6	61.9	75.5	83.1	69	268.6	133.1	108.2	393.2	52.5	23.3	19.3	64.1
Baselines													
All sleep	69.2	100	0	69.2	79.6	0	223.9	180.1	526.1	100	37.2	30.8	83.1
All wake	30.8	0	100	0	0	571.4	409.4	391.3	476.5	0	72.4	69.2	83.0

## Data Availability

The Newcastle Polysomnography dataset can be found at https://zenodo.org/record/1160410 (accessed on 15 October 2024). The MESA dataset can be found at https://sleepdata.org/datasets/mesa (accessed on 15 October 2024).

## Abbreviations

The following abbreviations are used in this manuscript:

AHI	Apnea–hypopnea index
CI	Confidence interval
CNN	Convolutional neural network
DACNN	Domain adversarial convolutional neural network
EEG	Electroencephalogram
MDPI	Multidisciplinary Digital Publishing Institute
DOAJ	Directory of open access journals
MAE	Mean average error
MESA	Multi-Ethnic Study of Atherosclerosis
PPG	Photoplethysmography
PSG	Polysomnography
RMSE	Root mean square error
ROC	Receiver operating characteristic
SDB	Sleep-disordered breathing
SE	Sleep efficiency
WASO	Wake after sleep onset
